# Gangrene*Read before the Massachusetts Veterinary Medical Association, October 1st, 1884.

**Published:** 1885-01

**Authors:** Joseph M. Skally


### Art. VI.—GANGRENE.*

BY JOSEPH M. SKALLY, V.S.

By gangrene is understood the death of an organ or part, as manifested by the more or less rapid breaking down and chemical decomposition of its texture. Gangrene may affect both soft and solid structures, the bones, for instance, or even fluids, as in necrosis or sepais of the blood. The breaking down of structures is generally a slow process, whilst in soft, juicy textures, and in fluids, it is rapidly consummated. Like normal textures, new formations of every kind, tumors, exudates, pus, are liable to become necrosed. Fluids degenerate through necrosis into gangrenous ichor, the most infectious and destructive of its tribe.

A general characteristic of gangrene is not easily given, so manifold are its forms, and so various are its causes. Soft parenchymata commonly breaks down into a diffluent pulp, marked by a high degree of discoloration and of fœtor. As decomposition proceeds, gases are generated in the parts, principally sulphuretted hydrogen, ammonia, nitrogen and carbonic acid. These give rise to the emphysematous crackling which is so often associated with the gangrenous processes. The tissues at the same time undergo a process of softening, or liquefaction, the limbs become exceedingly offensive, and owing to alterations in the transuded hæmoglobin, changes from a

* Read before the Massachusetts Veterinary Medical Association, October 1st, 1884.

reddish color to a brownish or greenish black. The characters of the dead parts vary with its structure, its vascularity, the cause of the gangrene, the acuteness of the process, and the possibility of the access of atmospheric air. The more vascular the part, the softer the structure, and the more it is exposed to the atmosphere, the more rapidly and completely does it undergo decomposition. Bones, cartilages, and tendons, which are firm, hard tissues, containing comparatively but few vessels, undergo very little alteration in structure and form; whereas softer parts are much more rapidly and completely destroyed. The occurrence of decomposition manifests itself in the first place in the blood contained in the part. The hæmoglobin escapes from the red blood corpuscles, partly by exudation, and partly by the destruction of the corpuscles themselves, and dissolved in the liquor sanguinea, permeates the surrounding tissues. The corpuscles are ultimately completely annihilated, nothing remaining but a few minute granules. The staining of the tissues with the hæmoglobin is known as *post mortem* staining, and the appearances it presents are very characteristic. All the tissues may be more or less affected, but the lining membranes of the heart and large blood-vessels, being in immediate contact with the blood after death, are naturally more so than other parts. The staining is of a uniform pinkish red color, thus differing from the puncti-form and strati-form redness of hyperæmia, from which it must be carefully distinguished. The amount of staining is in proportion to the rapidity with which the decomposition has taken place, and to the amount of blood contained in the part at the time of death. Gangrene has the import sometimes of a local, sometimes a symptom of general disease. The conditions necessary to the former case are nearly reducible to arrested afflux of blood—that is, stasis. It may begin by attacking fluid parts, and especially the blood, and extend from them to solid structures, or it may affect them all at once.

### DEVELOPMENT OF GANGRENE.

Gangrene is developed,

*First.*—Out of absolute blood stasis, which may occur under various circumstances.

(a). Every hyperæmia in organs, or sections of organs, particularly in paralyzed or enfeebled, or obnoxious to debilitating influences may degenerate into absolute stasis. This applies particularly to asthenic, hypostatic, hyperæmia in torpid peripherous organs, vegetating, so to say, imperfectly under the embarrassment of continued pressure.

(b). Mechanical hyperæmia frequently becomes absolute stasis as seen in incarcerated, strangulated organs, and as a result of the plugging of the returning vessels in the lower extremities.

(c). Every inflammatory stasis may degenerate into absolute stasis, more particularly those hypostatic and asthenic inflammations which occur in organs already diseased, paralyzed or depressed by violent external influences, such as concussion, contusion, heat or cold. An inflammation consequent upon influences directly or indirectly debilitating, may acquire during its progress, a tendency to absolute stasis. In absolute stasis, the blood undergoes gangrenous decomposition. Hence, the blood is the portion originally necrosed and dissolved. It exudes in a state of gangrenous ichors through the walls of the blood-vessels, causing the same gangrenous decomposition in these and in the surrounding tissues. This event gives rise to the most ordinary, and most developed form of moist gangrene. The progress of this gangrene is more or less acute, the gangrenous dissolution of tissues being marked by the rapidity of its course.

*Second.*—Gangrene is determined by failure in supply of blood.

(a). In impermeability of large arteries--high degree of calcification, and complete obstruction consequent upon arteritis and ossification.

Here gangrene takes the form of dry, black mummyfying gangrene.

(b). As a result of compression and tension of a part—as in strangulated hernia.

(c). As a result of local destruction of blood-vessels, the denudation of parts of attaching and blood supplying textures—bones, for example, of their external and internal periosteum, the common integuments of their supporting areolar tissue, the peritoneum of its subjacent layers, isolation of the pleura-pulmonalis over cavities of the lung. This gangrene appears as a white or yellowish-white slough.

(d). Extensive impermeability of the capillaries and minute vessels when plugged with coagula or compressed by surrounding exudates. In the last mentioned case, the gangrene is dependent upon inflammation. In this kind of gangrene, textures poor in blood-vessels, such as compact bones, callosities, etc., especially if brought into stasis or into coagulation, possess, in common with the exudates thrown out by it, an inherent tendency to gangrenous dissolution. It has already been stated that several varieties of gangrene have been recognized.

*Third.*—(1). Gangrene developed out of an internal cause is distinguished by the designation of primary gangrene, from that arising from an external cause.

(2). Hot, acute inflammatory gangrene, true gangrene, in the manner which inflammation leads to gangrene, is sufficiently clear from the foregoing.

(a). The inflammatory stasis, owing to its very intensity, to pre-existing debility of the diseased textures, or lastly to weakening influences caused during its progress, degenerated into absolute stasis.

(b). It occasions gangrene by the crushing effects of its products upon the capillaries, or by mechanical or ulcerous isolation of the textural parts. In this way gangrene may arise in tissues laboring under the sequelae of inflammation, without itself being an issue of the latter.

(3). Cold gangrene, sphacelus. This form is not in any way connected with inflammation.

*Fourth.*—Moist gangrene comprises the breaking down of fluid substances to gangrenous ichors and of fibrous textures to a various colored diffluent pulp, marked by the evolution of fœtid gases. It is the gangrene developed out of absolute blood stasis ; therefore, again inflammatory gangrene. It may be compared to the decomposition of animal matter under the co-operating influence of water.

*Fifth.*—Dry mummyfying and senile gangrene are the various terms designating this form of gangrene, which is caused by a deficient supply of blood. It manifests itself in the perishing of the implicated structures, with shrivelling or withering thereof to an incipiently tough, but eventually sloughing mass. Often, and particularly in the extremities, owing to impermeability of their arteries, the gangrenous textures blacken ; as such it is comparable to the decaying of organic matter with an insufficiency of moisture, and with the disengaging of pure carbon.

*Sixth.*—Black gangrene—gangrenous slough.

*Seventh.*—White gangrene occurs, for the most part, as a result of pressure in incarceration of the denuding of membranous expansions of their subjacent textures ; for example, as peritoneal sloughing at the base of intestinal ulcers. Of these different varieties of gangrene several are often present at the same time. Beneath the common integuments, often transformed into a swarthy, parched rind in senile gangrene, we often meet with patches of tissue which are reduced to humid, stinking pulp. Just as in gangrene of solids, gangrenous slough varies, so in like manner does gangrenous ichor, as necrosed blood or exudates vary, according to the crasis or constitution under which either has become attacked with gangrene. Like normal textures, diseased textures and new growths, fibroid, cancerous formations, for example, may become a prey to gangrene. Neither to ulceration or gangrene are all textures alike obnoxious. Bony, elastic, fibrous textures resist gangrene more ably than muscle. Areolar tissue, or mucous membrane, lax embryonic textures, like certain varieties of cancer, are especially prone to gangrenous destruction. The constituent elements of gangrenous texture masses are more or less well preserved textural debris, larger or smaller black contoured molecules, down to pulverulent granular mass, black pigment granules, fat drops and crystals. Contact with the atmosphere is by no means indispensable to the generation of gangrene. It affects equally with the external parts, organs never in contact with the air—as the liver and spleen. A very important phenomenon, involving a curative act, is the circumscription of gangrene by an inflammatory process of ulcerative isolation of this gangrenous part through its own secretion. The ultimate healing is brought about by the same inflammatory process changing to one of pus production and of regeneration. The organs most liable to be affected with gangrene in our practice (veterinary) are as follows, viz.: Peritoneum, stomach, intestine, liver, spleen, bladder, air passages, pleura, lungs, heart, vagina and uterus, penis, tongue, and bones. Of this last I shall not treat, leaving it to be disposed of under the common term applied to the disease, viz.: Necrosis of bone.

Gangrene of the peritoneum in the horse generally results from dividing the cord too high up, causing a high degree of erysipelatous fever, which usually extends to the peritoneum, which, along with the cord and surrounding structures, speedily becomes gangrenous. It may also be caused by pressure, as in hernia.

*Stomach.*—This is liable to become gangrenous through the introduction into it of the various acids in an undiluted state, and of the various caustic alkalies given in large doses by persons who are ignorant of their specific action. In reference to the intensity of the effect which may cause superficial or deep mortification of the tissues with greater or less rapidity, we distinguish several degrees. The effect is influenced by the quantity and the strength of the liquid and the duration of the period during which it remains in contact with the parts alluded to. We generally find the effect to be less intense in the cavity of the mouth and fauces, more marked in the œsophagus, and most powerful in the stomach. The entire mucous membrane is destroyed and converted into a black, soft mass, which is distended by a sanguinous fluid, and is easily detached from the muscular coats.

*Intestines.*—We find these affected by gangrene as a sequel to enteritis and intussusception, but as all these cases will prove fatal, I shall not dwell longer on them.

*Liver.*—Gangrene of this organ is of a very rare occurrence, but it is sometimes found associated with pulmonary gangrene. It is developed in parts affected with inflammation and suppuration. It occurs in more or less circumscribed spots, in which the paranchyma is dissolved into a brownish-black-green pulp, which diffuses the characteristic odor of sphacelus. We find suppuration in the vicinity, which is a product of reactive inflammation, and which defines the boundaries of the mortified parts.

*Spleen.*—Gangrene of the spleen is of as rare occurrence as the liver.

*Air passages.*—This affection occurs both here and in the parenchyma of the lung in two distinct forms, either as a circumscribed eschar on the mucous membrane, eating its way into the submucous tissue, in which it may occur primarily or as a diffuse gangrenous colliquescence of the bronchial mucous membrane. The conditions under which it is developed are similar to gangrene of the lung, with which it is sometimes combined. It generally, however, occurs in tissues in some way previously diseased, but appears rather as an accidental termination than as a necessary consequence of any peculiar local morbid process. It is most commonly associated with pulmonary gangrene.

*Pleura.*—Gangrene of the pleura occurs in consequence of its being denuded by accumulations of pus or ichor in the costal or pulmonary wall. The pleura then assumes the appearance of a yellowish-white, or more frequently of a blackish or greenish-brown lax or deliquescent slough, with superficial gangrene of the lung.

*Lung.*—Gangrene of the lungs is an affection of not infrequent occurrence, and under certain hepatization of a portion of a lung is a most common complication.

There are two distinct forms of gangrene of the lungs, viz.: diffuse and circumscribed or gangrenous eschar. In diffuse gangrene we find a portion of the lung presenting an abnormal greenish or brownish tint, filled with a similar colored, somewhat fatty, turbid serosity, soft, rotten, and breaking readily down, into a pulpy, shaggy tissue. The whole evolves the characteristic odor of sphacelus. Towards the outer portion the discoloration, infiltration and alteration of consistence are less marked, and finally become imperceptible, and there is no line of demarcation between the gangrenous and the adjacent tissue, which only differs from the normal state in being œdematous and ænemic. It corresponds to diffuse gangrene of the bronchial tubes, with which it is almost always associated. Upon the whole it is a rare affection, but when it does occur it always attains a considerable extent, as it commonly attacks one or more lobes. It is, perhaps, scarcely entitled to rank as an independent affection, as it is generally associated with eschar of the lungs, and hence it is more readily developed from the contact of the ichorous, gaseous and fluid products of the gangrenous eschar coming in contact with the bronchial and pulmonary mucous membrane, inasmuch as in all probability the disease extends from the bronchi to the lung tissue. The foregoing description of gangrene as it occurs in the upper lobes is sufficient to render this form intelligible, as well as to explain why there is no inflammatory reaction, and consequently no line of demarcation around the affected tissue. This form of gangrene very often follows as a sequel to fibrinous pneumonia. Circumscribed or partial gangrene of the lungs appears in the form of gangrenous eschar, and is more frequent than the former variety. We find the parenchyma at some spot of varying size converted into a blackish or brownish-green, hard but moist eschar, which adheres to the surrounding tissues, and evolving the peculiar odor of sphacelus, and similar to the eschar produced on the skin by nitrate of silver. It is sharply defined, and becomes gradually loosened from the surrounding tissues, and rests in a cavity corresponding to it in size and form. It may be described as a blackish-green plug, which is soft on the surface, with a firm centre floating in an ichorous fluid. More frequently the greater portion of the eschar softens and becomes dissolved into a greenish-brown fœtid ichorous pulp, mixed with rotten, shaggy fragments of tissue, and enclosed in a cavity, whose walls are lined by a shaggy tissue, infiltrated with ichor. These eschars may either occur singly, or several may be present. If the gangrenous eschar becomes detached it falls into the cavity of the thorax unless there be firm adhesions at the spot, or else it becomes dissolved, and the ichorous semi-solid matter is effused into the pleural sac, and gives rise to pleurisy with ichorous exudation, and to pneumothorax, since the fœtid gas evolved from the gangrenous mass either collects alone in the thorax, or atmospheric air finds its way out through the bronchial tubes which opens into the abscess, and thus mixes with the aforesaid gas in the thorax. Partial gangrene often arises in the perfectly healthy lungs of weak, decrepit and cachetic animals from general depressing influences, and is developed from a circumscribed passive stasis. Under simular circumstances we find it associated with pneumonia in its various stages, with pulmonary abscesses, with tuberculosis, with bronchitis, or it may be caused by the absorption of gangrenous ichor from gangrene of different parts into the blood.

*Heart.*—There is nothing at variance with the possibility of the occurrence of gangrene in the muscular substance of the heart. Ulcerations accompanied with malignant products are not of rare occurrence, but the correctness of the observations purporting to refer to gangrene of the heart have nevertheless been called in question by several writers ; and we must remark that no case of the kind ever came under our notice.

*Uterus.*—We sometimes find gangrene of the uterus, resulting from inversion, causing a gradual compression of the veins, the vessels become engorged with blood admitted to them faster than it can leave them, and so after intense congestion mortification ensues.

*Penis.*—We also meet with gangrene of the penis, resulting from paraphymosis or strangulation of the glans penis.

*Tongue.*—The point of this organ is, in rare cases, found in a gangrenous condition caused generally by the administration of some caustic irritant, such as turpentine, potash, etc., also by the pressure of the halter rope in leading, when it is passed through the mouth, enclosing the tongue.

When mortification has a tendency to spread, its dark color is gradually lost in the surrounding tissue, whereas, when it ceases to spread, a red line, called the line of demarcation, separates the dead from the living tissue. This line is always regarded as most important, indicating that sloughing has ceased to spread and that a process has begun for the removal of the sphacelated mass from the system. The final act in the separation of dead tissue is that of ulceration of portions of living tissue which are in immediate contact with the dead. A groove is formed by this ulceration which circumscribes and entrenches the dead part, and then gradually deepening and converging, undermines it until it reaches the centre, when the separation is complete and the slough falls off or is dislodged.

By the discharge of the ulcerated living tissue concomitant with this process of destruction one of repair is set up. As the ulcerated groove deepens so do granulation cells rise from its surface, so that one might say that which yesterday was ulcerating is granulating to-day, and thus very soon after the slough has separated the whole surface of the living part from which it was detached is covered with granulation and proceeds like an ordinary ulcer towards healing.

